# P155: Technologies to measure hand hygiene: examining the incorporation of the World Health Organisation (WHO) 5 moments

**DOI:** 10.1186/2047-2994-2-S1-P155

**Published:** 2013-06-20

**Authors:** C Dawson

**Affiliations:** 1 Institute of Digital Healthcare, University of Warwick, Coventry, UK

## Introduction

A case study of Hand Hygiene (HH) auditing within a UK NHS Acute Hospital resulted in the exploration of technology to improve efficiency and accuracy of HH measurement as per the evidence-based WHO 5 Moments of Hand Hygiene.

## Objectives

1. Determine the current state of measurement at case study site and identify perceived strengths/weaknesses.

2. Use WHO 5 Moments as benchmark to evaluate electronic monitoring (EM) technologies designed to capture HH compliance, and assess their perceived feasibility for use at case study site.

## Methods

Data was collected over 15 months. Observation and qualitative interviewing were used to produce a current state map of measurement. A structured literature review assessed Fit-For-Purpose of EM technologies. The current state map was used to assess their feasibility for use at case study site.

## Results

From a target pool of 124, 45 Healthcare Professionals (HP) were recruited including Infection Control, Nurses and Consultants; all involved in HH auditing; collecting data, receiving feedback or being subject to observation.

1. No explicit reference to WHO 5 Moments was included in current measurement of manual auditing using direct observation based upon standards for HH Technique (How) and Process (When).

Lack of clarity and consistency in content of audit feedback (AF) and the feedback process was identified as a weakness by all 30 participants (pp) interviewed.

A second weakness was lack of synergy between training content and content of AF data; training incorporates WHO 5 Moments yet AF contains no reference to performance at these times.

These weaknesses led the majority of pp (22/30) to conclude AF data is often ‘meaningless’.

2. No technologies able to monitor, measure and provide feedback on all 5 WHO Moments; 1, 4, 5 show potential for technology monitoring. HP are open to technology; all 13 ward based pp interested in potential for personal development and group improvement. Concerns raised in all interviews included ‘Big Brother’ culture and lack of Fit-for-Purpose tools.

## Conclusion

Weaknesses in current HH auditing processes can lead to perceptions of ‘meaningless’ data. No EM system currently fully supports the WHO 5 Moments, and whilst HP are open to technology, perceptions regarding use still exist and require addressing if EM systems are to be considered alternatives to direct observation.

## Disclosure of interest

None declared

